# Improving Deep Learning Based Lung Nodule Classification Through Optimized Adaptive Intensity Correction

**DOI:** 10.3390/bioengineering13040396

**Published:** 2026-03-29

**Authors:** Saba Khan, Muhammad Nouman Noor, Haya Mesfer Alshahrani, Wided Bouchelligua, Imran Ashraf

**Affiliations:** 1 Department of Artificial Intelligence and Data Science, National University of Computer and Emerging Sciences (FAST-NUCES), Islamabad 44000, Pakistannouman.noor@isb.nu.edu.pk (M.N.N.); 2 Department of Information Systems, College of Computer and Information Sciences, Princess Nourah Bint Abdulrahman University (PNU), P.O. Box 84428, Riyadh 11671, Saudi Arabia; halshahrane@pnu.edu.sa; 3 Applied College, Imam Mohammad Ibn Saud Islamic University (IMSIU), Riyadh 11432, Saudi Arabia; wabouchelligua@imamu.edu.sa; 4 Computer Engineering Lab, Quantum and Computer Engineering Department, EEMCS, TU Delft, 2628 CD Delft, The Netherlands

**Keywords:** lung nodule detection, computed tomography imaging, intensity normalization, contrast enhancement, deep learning, false positive reduction

## Abstract

Lung cancer is one of the most common causes of death from cancer around the world, and catching it early through computed tomography (CT) scans can drastically improve survival. However, automated classification of pulmonary nodule candidates is hard because images do not all have the same intensity across scanners and protocols, resulting in inconsistent performance, more false positives (FP), and a ceiling on how much deep learning models work in an average clinic. In this work, we tackle this by introducing a preprocessing step that corrects intensity differences before feeding images into classification models. We use Contrast-Limited Adaptive Histogram Equalization (CLAHE), but with its key parameters tuned automatically via a modified version of the Covariance Matrix Adaptation Evolution Strategy (CMA-ES). This helps to boost local contrast adaptively, keeps important anatomical details intact, and cuts down on noise. We tested the approach on the public LUNA16 dataset, first checking image quality (Peak Signal-to-Noise Ratio (PSNR) around 53 dB and Structural Similarity Index (SSIM) of 0.9, better than standard methods), then training three popular deep models—namely, ResNet-50, EfficientNet-B0, and InceptionV3—with CutMix augmentation for better generalization. On the enhanced images, ResNet-50 achieved up to 99.0% classification accuracy with substantially less FP than when using the raw scans. Taken together, these results demonstrate that intelligent and optimized preprocessing can effectively mitigate intensity variations via deep learning for lung nodule detection, thus coming closer to realizing the practical toolbox of computer-aided diagnosis in routine clinical practice.

## 1. Introduction

Lung cancer remains one of the leading causes of cancer-related mortality worldwide, imposing a substantial burden on healthcare systems and patients [1,2]. According to recent global estimates, approximately 2.48 million new cases and 1.82 million deaths are reported annually [3]. The high mortality rate is primarily attributed to late-stage diagnosis, as early-stage lung cancer often presents with subtle or asymptomatic findings [4]. Early detection significantly improves survival rates, increasing the likelihood of survival to over 50%, whereas late detection reduces survival to below 10% [5]. Pulmonary nodules observed in CT scans often represent the earliest indicators of lung cancer; however, their small size, low contrast, and visual similarity to benign structures such as blood vessels or airway walls make reliable detection challenging [6,7].

Low-dose computed tomography (LDCT) is currently the standard imaging modality for lung cancer screening, providing detailed volumetric information that enables early identification of nodules [8,9]. The widespread adoption of large-scale screening programs, such as the National Lung Screening Trial (NLST), has significantly increased the volume of CT scans analyzed in clinical practice. As a result, radiologists are required to review hundreds of slices per patient, increasing the risk of fatigue, inter-observer variability, and missed detections [2,10]. These challenges have motivated the development of computer-aided detection (CAD) systems based on artificial intelligence (AI), which assist radiologists by highlighting suspicious regions and improving diagnostic efficiency [11].

Deep learning approaches, particularly convolutional neural networks (CNNs), have demonstrated strong performance in lung nodule detection tasks [12,13]. Recent advancements, including attention mechanisms, transformer-based models, and hybrid architectures, have further improved detection sensitivity and contextual understanding [14,15]. Despite these advances, a critical limitation remains: models trained on controlled datasets often fail to generalize effectively to real-world clinical data [16]. One of the primary reasons for this performance gap is the variability in CT image intensity and contrast caused by differences in scanner types, acquisition protocols, reconstruction settings, and patient characteristics [17,18]. Since deep learning models rely heavily on intensity-based features, such variability can lead to unstable feature representations and degraded performance when applied to unseen data [19].

The impact of this intensity variability is clinically significant. Inconsistent contrast and noise patterns can cause benign anatomical structures to resemble pathological findings, increasing false positive detections and reducing diagnostic reliability [20,21]. Excessive false alarms also lead to unnecessary follow-up procedures and increased patient anxiety [22]. While recent studies have focused on improving model architectures, these approaches often assume that input images are already well-normalized. In practice, this assumption does not hold, and architectural complexity alone cannot compensate for inconsistent or low-quality input data. Consequently, recent literature emphasizes the importance of preprocessing and intensity harmonization as critical steps for improving robustness in medical image analysis [23].

Motivated by these observations, this study focuses on improving model robustness at the input level rather than introducing a new detection architecture. We propose an adaptive intensity correction framework that mitigates scanner- and protocol-induced variability prior to deep learning analysis. The framework is based on contrast-limited adaptive histogram equalization (CLAHE), a widely used technique for enhancing local contrast in medical images [24,25]. However, the effectiveness of CLAHE is highly dependent on parameter selection, and manual tuning often fails to generalize across datasets [26]. To address this limitation, CLAHE parameter selection is formulated as an optimization problem, and CMA-ES is employed with a task-specific composite objective function to automatically determine optimal parameters. The enhanced images are subsequently used to train deep learning models, including ResNet-50, EfficientNet-B0, and InceptionV3, with CutMix augmentation to improve generalization [27,28].

It is important to distinguish between lung nodule detection and lung nodule classification tasks. Lung nodule detection refers to the localization of suspicious regions within full CT volumes, whereas classification focuses on determining whether a given candidate region corresponds to a nodule or non-nodule. In this study, we adopt a candidate-based classification framework using pre-extracted regions from the LUNA16 dataset. Therefore, the primary focus of this work is lung nodule classification rather than end-to-end detection.

The main contributions of this work are summarized as follows:

- We propose an adaptive intensity correction pipeline based on CMA-ES-optimized CLAHE with a task-specific objective function to reduce scanner- and protocol-induced variability in lung CT images, achieving improved image quality (PSNR ≈53dB, SSIM ≈0.9).
- We demonstrate that improved intensity consistency reduces false positive detections by enhancing feature reliability and minimizing confusion between benign and malignant structures.
- We validate the proposed framework on the LUNA16 dataset, achieving up to 99.0% classification accuracy with ResNet-50, demonstrating improved robustness without increasing model complexity.

Overall, the proposed approach highlights the importance of optimized preprocessing in enhancing the reliability of AI-based lung cancer detection systems and provides a promising direction for improving performance under heterogeneous imaging conditions, with potential for future clinical applicability.

## 2. Related Work

Research on lung nodule analysis in computed tomography (CT) has evolved from handcrafted pipelines to end-to-end deep learning frameworks. Earlier approaches typically combined thresholding, morphological operations, and radiomic feature extraction with conventional classifiers such as support vector machines (SVMs). Although these methods offered some interpretability, their dependence on manually engineered features limited their ability to capture subtle variations in nodule appearance and reduced generalization across datasets [29].

With the rise of deep learning, convolutional neural networks (CNNs) became the dominant paradigm for lung nodule detection and classification. Early 2D CNNs processed individual slices or small patches and improved feature learning compared with traditional methods, but they often lacked sufficient volumetric context and produced elevated false positive rates [12,30]. Later, 3D CNNs and multi-scale volumetric models addressed this limitation by incorporating spatial continuity across slices, leading to improved sensitivity and more reliable detection performance [7,13]. Transfer learning with ImageNet-pretrained backbones such as ResNet, Inception, and EfficientNet further improved convergence and performance in settings with limited annotated medical data [27,31]. More recently, hybrid CNN-based models, attention mechanisms, and transformer architectures have been introduced to better capture global context and small nodule characteristics [15,23,32].

Despite these architectural advances, a major challenge remains unresolved: the variability in CT intensity and contrast across scanners, reconstruction kernels, acquisition protocols, and patient conditions. Such variability can substantially affect the robustness of deep learning models, especially when applied to images acquired under conditions different from those seen during training [16,17,19]. Consequently, preprocessing and intensity harmonization have become important components of lung CT analysis pipelines.

### 2.1. Intensity Correction and Enhancement Techniques

Existing intensity correction techniques for lung CT imaging range from simple global normalization methods to more adaptive and learning-based approaches. Global histogram equalization and standard normalization methods improve overall contrast but often amplify noise and non-nodular structures, limiting their usefulness in low-contrast regions [29,33]. Locally adaptive approaches, particularly contrast-limited adaptive histogram equalization (CLAHE), have received considerable attention because they enhance local contrast and improve the visibility of subtle nodules [33,34]. However, their performance is highly dependent on parameter selection, and inappropriate settings may introduce artifacts or over-enhancement [26,34].

To address noise and low-dose imaging issues, some studies combine contrast enhancement with denoising filters such as Gaussian or adaptive median filtering [35]. More advanced approaches, including bias field correction and GAN-based harmonization, have also been explored to reduce scanner-induced variability [19,36,37]. While these methods are promising, they may increase computational complexity or introduce anatomically unreliable intensity transformations. Overall, the literature indicates that adaptive enhancement is useful, but robustness still depends strongly on careful parameter control. This motivates the use of optimization-based strategies for determining enhancement parameters automatically.

Table 1 summarizes representative intensity correction methods reported in the literature.

### 2.2. Model Architectures for Lung Nodule Detection

Model development in lung nodule analysis has progressed from radiomics-based classifiers to deep neural architectures capable of learning hierarchical representations directly from CT images. Classical CAD systems using handcrafted features and SVMs were limited by low flexibility and weak generalization [29]. CNN-based methods substantially improved performance by learning discriminative texture and shape features automatically [12]. In particular, 3D CNNs improved sensitivity by exploiting volumetric context, although their computational cost and risk of overfitting remain important limitations [7,38].

Transfer learning and lightweight architectures such as ResNet-50, InceptionV3, and EfficientNet have become common due to their strong performance and practicality [27,33]. In parallel, attention-based and hybrid models have been proposed to better suppress irrelevant regions and improve detection of small nodules [32,39,40]. Transformer-based methods have also shown promise for modeling long-range contextual dependencies, but they typically require larger datasets and greater computational resources [15,23]. Importantly, several studies report that even strong architectures remain sensitive to intensity inconsistency across scanners, highlighting that architectural refinement alone is insufficient for robust deployment [16,17].

Table 2 provides a concise comparison of representative lung nodule detection models.

### 2.3. False Positive Reduction (FPR)

False positive reduction is a critical requirement in lung nodule CAD systems because excessive false alarms increase radiologists’ workload and reduce trust in automated tools. Early deep learning systems often achieved acceptable sensitivity only at the cost of multiple FP per scan [6,10]. To address this issue, two-stage pipelines became common, where an initial detector maximizes sensitivity and a second-stage classifier removes non-nodular candidates. Later studies demonstrated that incorporating volumetric context, candidate refinement, and attention mechanisms can substantially reduce FP while maintaining high sensitivity [32,45,46,47].

Although recent methods report strong false positive reduction on benchmark datasets, their performance may degrade when applied to unseen intensity conditions or heterogeneous clinical data [17,32]. This suggests that false positive reduction depends not only on model design but also on the consistency and reliability of the input images.

Table 3 summarizes representative false positive rates reported in previous studies.

### 2.4. Summary of Research Gap

In summary, prior work shows that lung nodule detection performance has improved substantially through deep learning, volumetric modeling, and attention-based architectures. Similarly, preprocessing methods such as CLAHE and related enhancement techniques have been shown to improve visual quality and feature visibility. However, three important limitations remain. First, many studies rely on fixed or manually tuned enhancement settings, which may not generalize across datasets. Second, strong model architectures still remain sensitive to scanner- and protocol-induced intensity variability. Third, false positive reduction is often studied at the architectural level without sufficiently addressing instability in the input images.

These limitations motivate the present work, which focuses on adaptive intensity correction through optimization-driven CLAHE parameter selection before classification. By improving intensity consistency prior to deep learning analysis, the proposed framework aims to enhance robustness, reduce FP, and improve generalization without increasing architectural complexity.

## 3. Methodology

**Figure 1:**
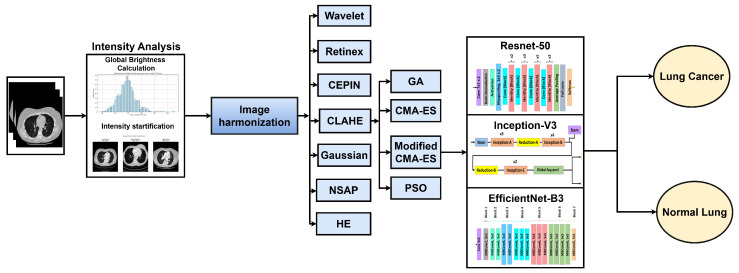
Flowchart of the proposed research methodology for automated lung cancer diagnosis.

This section presents the workflow of the proposed framework, illustrated in Figure 1. The pipeline consists of five stages: dataset preparation, intensity analysis, image enhancement, data augmentation, and deep learning-based classification. The aim is to reduce scanner- and protocol-induced intensity variability before model training and then evaluate the effect of the proposed preprocessing on lung nodule classification.

To ensure fair evaluation and reduce overfitting, the dataset was divided into three mutually exclusive subsets: training (70%), validation (10%), and testing (20%). The scan distribution is summarized in Table 4.

### 3.1. Data Loading and Candidate Extraction

Experiments were performed on the LUNA16 dataset, a standardized subset of LIDC-IDRI widely used for lung nodule analysis [6]. The dataset contains 888 chest CT scans stored in .mhd/.raw format, preserving voxel-level Hounsfield Unit (HU) information required for intensity-based preprocessing [8].

Ground-truth nodules were obtained from the LUNA16 annotations file, while candidate nodule and non-nodule locations were obtained from the candidates file [6]. Since candidate coordinates are provided in world coordinates, each location was converted into voxel indices using the scan origin and spacing from the corresponding .mhd header [8]. Candidate-centered regions of interest (ROIs) were then extracted for subsequent analysis and classification.

**Figure 2:**
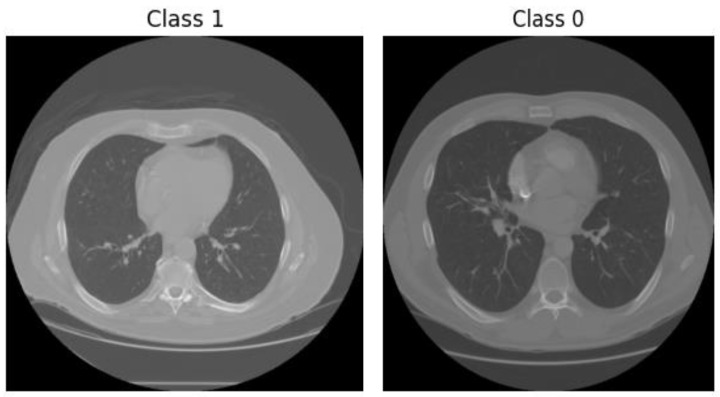
Examples of nodule and non-nodule candidate regions extracted from the LUNA16 dataset.

Because non-nodule candidates largely outnumber nodule candidates, controlled negative sampling and class balancing were used during sample preparation to avoid bias toward the majority class [48]. Representative samples were visually verified to ensure correct coordinate mapping and patch extraction. From the candidates file, which contains more than 550,000 candidate locations, a balanced subset was constructed for training and evaluation. All positive nodule candidates were retained, while negative samples (non-nodules) were randomly selected to achieve a balanced or near-balanced class distribution.The same candidate selection strategy and dataset splits were consistently applied across all experiments to ensure fair comparison and reproducibility. This controlled sampling approach prevents bias toward the majority class and ensures that the models are trained on representative and diagnostically relevant samples. Representative examples of extracted candidates are shown in Figure 2.

### 3.2. Intensity Analysis

To characterize intensity variability across scans, average lung-region intensity was computed after restricting analysis to the lung parenchyma using lung masks [49]. For each scan, the average lung intensity was calculated as

(1) $$I_{\mathrm{avg}}=\frac{1}{N}\sum_{i=1}^{N}P_{i},$$

where *N* is the number of pixels inside the lung mask and $P_{i}$ is the HU value of the *i*-th lung pixel. Based on these values, scans were grouped into low-, medium-, and high-intensity categories to examine scanner-induced appearance differences and evaluate robustness across varying intensity conditions [50].

**Figure 3:**
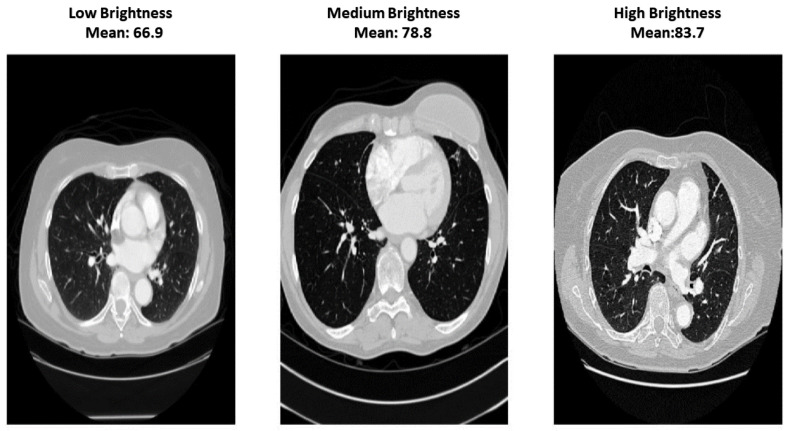
Comparison of CT scan intensity levels categorized into Low, Medium, and High brightness groups.

This stratification is illustrated in Figure 3 and provides a simple way to assess whether enhancement reduces inter-scan variability and improves consistency across different intensity ranges.

### 3.3. Image Enhancement Techniques

**Figure 4:**
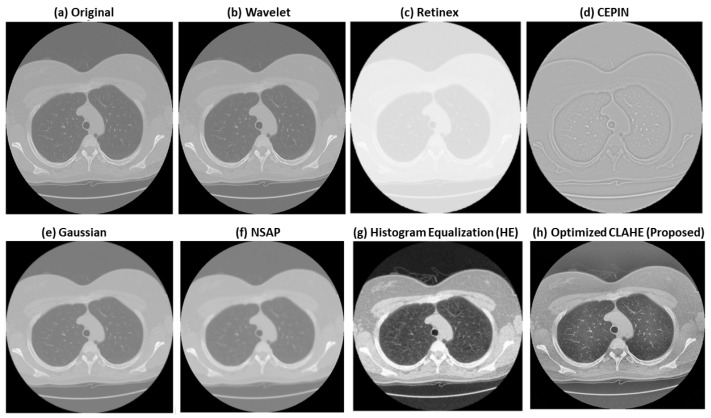
Visual comparison of the investigated image enhancement techniques on a representative CT slice: (**a**) Original, (**b**) Wavelet, (**c**) Retinex, (**d**) CEPIN, (**e**) Gaussian, (**f**) NSAP, (**g**) Histogram Equalization (HE), and (**h**) Optimized CLAHE (Proposed). The compared methods show different trade-offs between contrast enhancement, noise suppression, and structural preservation. The proposed optimized CLAHE provides the most balanced visual improvement, with clearer local contrast and better preservation of anatomical detail.

Several enhancement methods were evaluated to improve local contrast and suppress visually irrelevant noise before classification. These included Wavelet-based enhancement, Retinex, Contrast Enhancement via Pixel Intensity Normalization (CEPIN), Gaussian filtering, Noise Suppression Adaptive Processing (NSAP), Histogram Equalization (HE), and the proposed optimized CLAHE. These methods were selected as representative global, local, denoising, and normalization-based approaches commonly used in medical image preprocessing. A visual comparison of these enhancement techniques is shown in Figure 4.

Wavelet enhancement was used to emphasize fine-scale structural details and edges [51,52]. Retinex was included as an illumination-correction approach for improving brightness consistency [16,52]. CEPIN was used as an intensity normalization technique to reduce inter-scan variation [17,25]. Gaussian filtering and NSAP were evaluated as representative smoothing methods for noise reduction, with NSAP intended to preserve more structural detail than standard Gaussian filtering [53]. Histogram Equalization was included as a conventional global contrast enhancement baseline [52,54].

These methods were used mainly for comparative evaluation rather than as the final preprocessing choice. Based on both visual inspection and quantitative assessment, optimized CLAHE produced the most balanced improvement in local contrast, structural preservation, and artifact control. Therefore, it was selected for the proposed framework.

### 3.4. CLAHE Optimization Strategy

CLAHE was adopted because it enhances local contrast while limiting the over-amplification of noise that is commonly observed in standard adaptive histogram equalization [24,25]. In lung CT images, this is particularly important because subtle nodules must be enhanced without distorting surrounding tissue structures.

Let the input image be represented as

(2) I(x,y),x∈[1,H],y∈[1,W],

where the image is divided into local tiles. For each tile, a histogram is computed and clipped using a clip limit *C*:

(3) $$\tilde{h}\left(i\right)=\min\left(h(i),C\right).$$

The cumulative distribution function is then calculated as

(4) $$\mathrm{CDF}\left(i\right)=\frac{1}{N}\sum_{j=0}^{i}\tilde{h}\left(j\right),$$

where *N* denotes the number of pixels in the tile. Bilinear interpolation is applied between neighboring tiles to avoid boundary artifacts.

The two key CLAHE parameters are the clip limit *C* and tile size *T*. Since manual parameter selection is not robust across scans and imaging conditions [26], parameter tuning was formulated as an optimization problem:

(5) θ={C,T},

(6) $$\theta^{*}=\arg\max_{\theta }f\left(\theta \right).$$

To optimize θ, both PSO and CMA-ES were explored, while the final framework uses a CMA-ES with a task-specific composite objective function due to its better balance of image fidelity and structural preservation.

#### CMA-ES with Composite Objective Function

In standard CMA-ES, candidate parameter sets are sampled from a multivariate normal distribution:

(7) $$\theta_{k}∼N\left(\mu ,\Sigma \right),$$

where μ is the mean parameter vector and Σ is the covariance matrix. To better preserve diagnostically relevant structures, a composite objective function was defined as

(8) f(θ)=αPSNR(θ)+βSSIM(θ)−γNRMSE(θ),

where PSNR and SSIM reward image fidelity and structural consistency, while NRMSE penalizes distortion [55]. This formulation encourages enhancement settings that improve local contrast without introducing unrealistic textures.

The search space was defined as

(9) C∈[0.01,0.5],T∈{8,16,32,64}.

Since only two CLAHE parameters are optimized, the computational burden remains moderate and is incurred offline only once before model training and inference. The optimized CLAHE parameters are then applied uniformly to all CT images, resulting in negligible preprocessing overhead during deployment. It should be noted that the CMA-ES algorithm itself is not modified. Instead, the novelty lies in the formulation of a task-specific composite objective function tailored for lung CT enhancement.

The optimization of CLAHE parameters using CMA-ES is performed offline prior to model training and inference. The computational cost depends on the population size, number of generations, and repeated evaluation of the objective function based on PSNR, SSIM, and NRMSE. However, since only two parameters (clip limit and tile size) are optimized, the overall computational burden remains moderate.

Once optimal parameters are obtained, the same configuration is applied to all CT images. Therefore, the preprocessing step introduces negligible overhead during inference. The test-time computational cost is dominated by the forward pass of the deep learning model.

### 3.5. Data Augmentation Using CutMix

To improve generalization and reduce overfitting, CutMix augmentation was applied during training [28,56]. Given two training images $x_{A}$ and $x_{B}$ with labels $y_{A}$ and $y_{B}$, the augmented image is defined as

(10) $$\tilde{x}=M⊙x_{A}+\left(1-M\right)⊙x_{B},$$

where *M* is a binary mask defining the replaced region. The corresponding soft label is computed as

(11) $$\tilde{y}=\lambda y_{A}+\left(1-\lambda \right)y_{B},$$

with

(12) $$\lambda =\frac{\mathrm{Area}(M)}{H\times W}.$$

During training, λ was sampled from a Beta distribution:

(13) λ∼Beta(α,α).

CutMix was used to expose the models to more diverse spatial contexts and to reduce reliance on small highly discriminative regions, thereby improving robustness.

### 3.6. Model Development and Validation

Three transfer learning models were investigated: ResNet-50, InceptionV3, and EfficientNetB0 [27]. These architectures were selected because they represent complementary design strategies: residual learning, multi-scale feature extraction, and efficient compound scaling.

All networks were initialized with ImageNet-pretrained weights and fine-tuned on the enhanced CT images [31]. The classification task was binary, distinguishing nodule from non-nodule candidates. For fair comparison, all models used the same train/validation/test split and the same preprocessing pipeline.

#### 3.6.1. ResNet-50

ResNet-50 is a deep convolutional neural network based on residual learning, where shortcut (skip) connections are introduced to alleviate the vanishing gradient problem and enable stable training of very deep architectures [57]. Instead of learning a direct mapping, the network learns residual functions, allowing efficient feature propagation and improved convergence.

The architecture consists of multiple residual blocks with bottleneck structures, where each block includes 1×1, 3×3, and 1×1 convolutions. These bottleneck layers reduce computational complexity while maintaining strong representational power. This design allows ResNet-50 to capture hierarchical features ranging from low-level edges to high-level semantic structures, which are critical for distinguishing pulmonary nodules from surrounding tissues.

**Figure 5:**
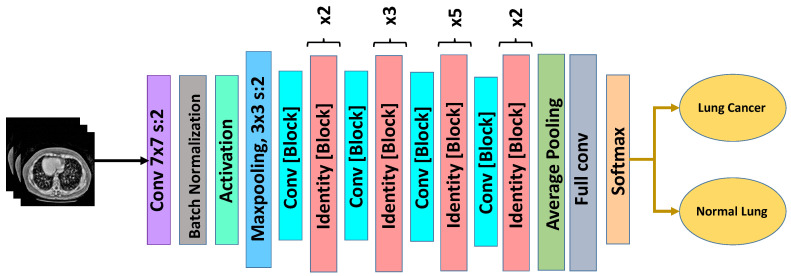
ResNet-50 architecture used for lung cancer classification.

The architecture of the adopted ResNet-50 model is illustrated in Figure 5. In this study, the ImageNet-pretrained ResNet-50 backbone was fine-tuned on the enhanced CT images. The original classification head was removed and replaced with a global average pooling layer followed by a fully connected dense layer with sigmoid activation for binary classification (nodule vs. non-nodule).

The model was trained using binary cross-entropy loss and the Adam optimizer with a learning rate of $1\times 10^{-4}$. Dropout regularization was applied in the final layers to reduce overfitting and improve generalization.

Due to its residual learning mechanism, ResNet-50 demonstrates strong robustness to intensity variations and noise compared with other architectures. When combined with the proposed optimized CLAHE preprocessing, the model achieves superior feature discrimination and classification performance, as demonstrated in the experimental results.

#### 3.6.2. InceptionV3

InceptionV3 captures multi-scale features through parallel convolutional paths with different kernel sizes, making it suitable for nodules with varying sizes and appearances [15,30]. This architecture employs Inception modules that factorize convolutions into smaller operations (e.g., 1×1, 3×3, and asymmetric convolutions such as 1×3 followed by 3×1), which improves computational efficiency while preserving representational capacity.

In addition, InceptionV3 incorporates batch normalization and auxiliary classifiers to stabilize training and mitigate vanishing gradient issues. These design choices enable the model to capture both fine-grained local textures and broader contextual patterns, which are essential for distinguishing small pulmonary nodules from surrounding anatomical structures.

**Figure 6:**
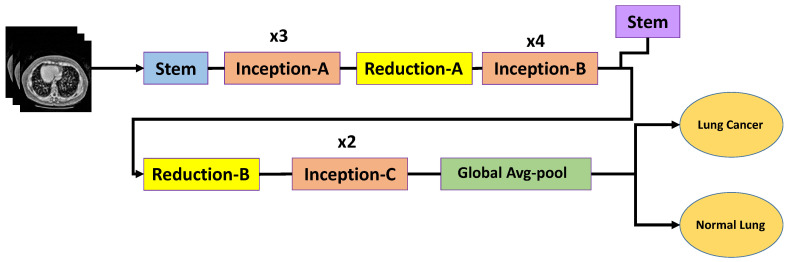
InceptionV3 architecture for lung cancer classification.

The architecture of the adopted InceptionV3 model is illustrated in Figure 6. In this study, the ImageNet-pretrained InceptionV3 backbone was fine-tuned on the enhanced CT images. The original classification head was removed and replaced with a global average pooling layer followed by a fully connected dense layer with sigmoid activation for binary classification (nodule vs. non-nodule). Dropout regularization was also applied to reduce overfitting.

Despite its strong multi-scale feature extraction capability, InceptionV3 is relatively sensitive to intensity variations and noise in raw CT images. However, after applying the proposed optimized CLAHE preprocessing, the model demonstrates improved feature discrimination and classification performance, as shown in the experimental results.

#### 3.6.3. EfficientNetB0

EfficientNetB0 is a lightweight convolutional neural network that achieves an optimal balance between accuracy and computational efficiency through compound scaling of network depth, width, and input resolution [27]. Unlike conventional architectures that scale these dimensions independently, EfficientNet uniformly scales all three using a fixed scaling coefficient, resulting in improved performance with fewer parameters.

The architecture is based on mobile inverted bottleneck convolution (MBConv) blocks combined with squeeze-and-excitation (SE) modules, which enhance channel-wise feature recalibration and improve the network’s ability to focus on relevant anatomical structures. These design characteristics make EfficientNetB0 particularly suitable for medical imaging tasks where subtle texture variations and small structures must be accurately captured under computational constraints.

**Figure 7:**
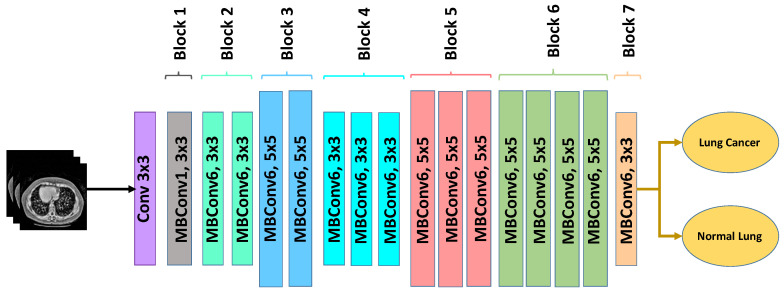
EfficientNetB0 architecture for lung cancer classification.

The architecture of the adopted EfficientNetB0 model is illustrated in Figure 7. In this study, the ImageNet-pretrained EfficientNetB0 backbone was fine-tuned on the enhanced CT images. The original classification head was replaced with a global average pooling layer followed by a fully connected dense layer with sigmoid activation for binary classification (nodule vs. non-nodule). Dropout regularization was applied before the final dense layer to improve generalization and reduce overfitting.

The model was trained using binary cross-entropy loss and the Adam optimizer with consistent hyperparameters across all architectures. Due to its compact design, EfficientNetB0 offers faster training and inference compared with deeper models while maintaining competitive classification performance. After applying the proposed optimized CLAHE preprocessing, the model demonstrates significant improvement in sensitivity, indicating enhanced capability to detect subtle and low-contrast pulmonary nodules.

All models were trained under identical experimental settings, enabling a fair comparison of classification performance after applying the proposed intensity-correction framework.

## 4. Results and Discussion

### 4.1. Experimental Setup

This section describes the dataset used, data partitioning strategy, computational environment, implementation details, training configuration, and evaluation metrics employed for assessing both image enhancement quality and lung nodule classification performance. A standardized and reproducible experimental protocol is adopted to ensure fair comparison across enhancement techniques and deep learning architectures.

#### Dataset and Data Splits

All experimental results reported in this section are obtained using the LUNA-16 dataset and the train/validation/test partitioning described in Section 3. The same scan-wise, patient-independent split of 70% for training, 10% for validation, and 20% for testing is maintained for all experiments to ensure fair and consistent comparison across different models and preprocessing configurations.

### 4.2. Implementation Details

All experiments were conducted on a high-performance workstation specifically configured to support large-scale deep learning training and evaluation. The hardware platform consisted of an Intel Core i9 processor, 64 GB of system memory, and an NVIDIA RTX 3090 graphics processing unit equipped with 24 GB of dedicated VRAM. This configuration provides sufficient computational capability to handle high-resolution CT images, extensive data augmentation, and fine-tuning. The operating system used was Ubuntu 20.04, which ensures stable compatibility with modern GPU drivers and deep learning libraries.

On the software side, all implementations were carried out using Python 3.8. The deep learning models were developed using the TensorFlow 2.x framework with the Keras high-level API, which offers flexibility for transfer learning, fine-tuning, and custom model design. CUDA 11.x and cuDNN 8.x were utilized to enable GPU acceleration and efficient parallel computation. Image preprocessing, enhancement, and augmentation operations were performed using the OpenCV and NumPy libraries, which are widely adopted for medical image processing tasks.

To ensure fair comparison and reproducibility of results, several standard experimental control measures were applied. Random seeds were fixed for NumPy and TensorFlow to reduce stochastic variations in weight initialization and data shuffling. Where supported by the framework, deterministic GPU operations were enabled to further stabilize training behavior. In addition, the same training, validation, and testing splits were used consistently for all models and experiments, ensuring that observed performance differences are attributable solely to the proposed preprocessing and modeling strategies rather than dataset partitioning effects.

The complete hardware and software environment used in this study is summarized in Table 5.

### 4.3. Training Configuration

All deep learning models—namely, ResNet-50, EfficientNet-B0, and InceptionV3—are trained using a transfer learning paradigm in order to leverage knowledge learned from large-scale natural image datasets. Specifically, weights pretrained on the ImageNet dataset are employed to initialize each network, providing robust low-level feature representations such as edges, gradients, and textures. During the initial training stage, early convolutional layers are frozen to preserve these generic features, while deeper layers are gradually unfrozen and fine-tuned to adapt the networks to domain-specific characteristics of lung CT images. This strategy accelerates convergence, stabilizes training, and mitigates overfitting, particularly in scenarios with limited annotated medical data.

All input CT patches are resized to a spatial resolution of 224×224 pixels, which matches the default input requirements of the selected backbone architectures. Training is performed with a batch size of 16, balancing GPU memory usage and gradient stability. The Adam optimizer is employed due to its adaptive learning rate capabilities and proven effectiveness in medical imaging applications. The initial learning rate is set to $1\times 10^{-4}$, and model optimization is guided using the binary cross-entropy loss function, which is appropriate for the binary classification task of nodule versus non-nodule.

To avoid overfitting and improve generalization, several regularization strategies are incorporated. A dropout rate of 0.5 is applied in the fully connected layers to randomly deactivate neurons during training. Furthermore, early stopping with a patience of 20 epochs is utilized, whereby training is halted if the validation loss fails to improve, ensuring selection of the best-performing model checkpoint. Additionally, a ReduceLROnPlateau learning rate scheduling strategy is adopted, reducing the learning rate by a factor of 0.5 when validation loss plateaus for 10 consecutive epochs. Because lung nodule datasets are inherently imbalanced, with non-nodule samples significantly outnumbering nodules, class imbalance is addressed through a combination of class weighting and controlled negative sampling. These measures prevent bias toward the majority class and encourage the models to learn discriminative features for malignant nodules. A summary of the main training hyperparameters used across all experiments is provided in Table 6.

During training, CutMix data augmentation is applied online by randomly combining regions from pairs of training images along with proportionally mixed labels. This strategy encourages the networks to learn from mixed semantic contexts, reduces reliance on small discriminative regions, and improves robustness.

### 4.4. Evaluation Metrics

To comprehensively assess the effectiveness of the proposed framework, two complementary groups of evaluation metrics are employed. The first group focuses on objective image quality assessment to quantify the impact of enhancement techniques on CT images, while the second group evaluates classification performance for lung nodule detection. Together, these metrics provide a balanced analysis of both preprocessing quality and diagnostic reliability.

#### 4.4.1. Image Enhancement Metrics

Objective image quality evaluation is essential to verify that enhancement techniques improve visual clarity without introducing distortion or artificial structures. Four widely adopted quantitative metrics are used for this purpose.

- Peak Signal-to-Noise Ratio (PSNR): PSNR measures the fidelity of the enhanced image relative to the original image and reflects the amount of noise or distortion introduced during enhancement. Higher PSNR values indicate better reconstruction quality and minimal degradation. It is defined as
  (14) $$\mathrm{PSNR}=10\log_{10}\left(\frac{MAX_{I}^{2}}{\mathrm{MSE}}\right),$$
  where $MAX_{I}$ denotes the maximum possible pixel intensity value. Here, $\log_{10}$ denotes the base-10 logarithm.
- Structural Similarity Index (SSIM): SSIM evaluates perceptual similarity by comparing luminance, contrast, and structural information between the original and enhanced images. Unlike pixel-wise error metrics, SSIM correlates well with human visual perception. Its value ranges from 0 to 1, with values closer to 1 indicating superior structural preservation.
- Mean Squared Error (MSE): MSE measures the average squared difference between pixel intensities of the original image *I* and the enhanced image $\hat{I}$:
  (15) $$\mathrm{MSE}=\frac{1}{N}\sum_{i=1}^{N}{\left(I_{i}-{\hat{I}}_{i}\right)}^{2},$$
  where *N* is the total number of pixels. Lower MSE values indicate reduced pixel-level distortion.
- Normalized Root Mean Square Error (NRMSE): NRMSE is a scale-independent version of RMSE and provides a normalized measure of reconstruction error:
  (16) $$\mathrm{NRMSE}=\frac{\sqrt{\mathrm{MSE}}}{I_{\max}-I_{\min}}.$$
  Lower NRMSE values indicate better enhancement quality with minimal intensity deviation.

In the context of lung CT imaging, high PSNR and SSIM values are desirable to ensure preservation of anatomical structures such as pulmonary nodules, vessel boundaries, and parenchymal textures, while low MSE and NRMSE confirm controlled enhancement without introducing artifacts.

#### 4.4.2. Classification Metrics

To evaluate lung nodule classification performance, several standard diagnostic metrics are reported. These metrics capture both overall accuracy and clinically relevant error behavior.

Let TP, TN, FP, and FN denote true positives, true negatives, FP, and false negatives, respectively.

Accuracy:

(17) $$\mathrm{Accuracy}=\frac{\mathrm{TP}+\mathrm{TN}}{\mathrm{TP}+\mathrm{TN}+\mathrm{FP}+\mathrm{FN}}.$$

Precision:

(18) $$\mathrm{Precision}=\frac{\mathrm{TP}}{\mathrm{TP}+\mathrm{FP}}.$$

Recall (Sensitivity):

(19) $$\mathrm{Recall}=\frac{\mathrm{TP}}{\mathrm{TP}+\mathrm{FN}}.$$

Specificity:

(20) $$\mathrm{Specificity}=\frac{\mathrm{TN}}{\mathrm{TN}+\mathrm{FP}}.$$

F1-Score:

(21) $$F1=2\times \frac{\mathrm{Precision}\times \mathrm{Recall}}{\mathrm{Precision}+\mathrm{Recall}}.$$

Area Under the ROC Curve (AUC): AUC measures the model’s ability to discriminate between nodule and non-nodule classes across varying decision thresholds. Higher AUC values indicate stronger class separability and robustness.

From a clinical perspective, recall and false negative behavior are particularly critical, as missed malignant nodules can delay diagnosis and treatment. Therefore, the classification results are interpreted with special emphasis on sensitivity and AUC rather than accuracy alone.

### 4.5. Quantitative Evaluation of Image Enhancement

This subsection evaluates the effectiveness of the investigated image enhancement techniques using objective image quality metrics. Because pulmonary nodule detection relies heavily on local contrast, edge clarity, and structural fidelity, it is essential to quantitatively verify that enhancement operations improve visual quality without introducing distortion [18]. Four widely adopted objective metrics are employed: Peak Signal-to-Noise Ratio (PSNR), Structural Similarity Index (SSIM), Mean Squared Error (MSE), and Normalized Root Mean Square Error (NRMSE). Collectively, these metrics assess noise suppression, contrast fidelity, and structural preservation in enhanced CT images [58].

#### 4.5.1. Objective Image Quality Metrics

PSNR measures the ratio between the maximum possible signal power and the power of distortion introduced by enhancement. Higher PSNR values indicate better reconstruction fidelity and reduced noise. SSIM evaluates perceptual similarity between the original and enhanced images by considering luminance, contrast, and structural consistency; values closer to one indicate superior structural preservation. MSE and NRMSE quantify pixel-wise reconstruction error, where lower values correspond to less distortion.

Together, high PSNR and SSIM combined with low MSE and NRMSE indicate an enhancement method that improves contrast while preserving anatomical integrity—an essential requirement for lung CT analysis [59].

#### 4.5.2. Comparison of Enhancement Methods

A quantitative comparison of all investigated enhancement techniques is reported in Table 7. The evaluated methods include Wavelet-based enhancement, Retinex, CEPIN, Gaussian filtering, NSAP, Histogram Equalization (HE), CLAHE with different optimizers, and the proposed CMA-ES with a task-specific composite objective function with optimized CLAHE [24].

It is important to note that PSNR values are computed with respect to the original CT images rather than ground-truth references. Since CLAHE primarily redistributes intensity values without introducing strong distortions, this can lead to higher PSNR values compared to reconstruction-based tasks. Similar trends have been observed in prior enhancement studies.

Traditional global enhancement techniques such as Retinex and HE exhibit relatively low PSNR and SSIM values and very high MSE and NRMSE, indicating that these methods introduce considerable distortion and fail to preserve fine anatomical details. Although Retinex attempts to correct illumination variations, it tends to amplify noise in homogeneous lung regions, making it unsuitable for reliable CT preprocessing. Wavelet-based enhancement achieves strong structural preservation, reflected by a high SSIM (0.98), but its limited local contrast adaptation results in lower PSNR compared with advanced CLAHE-based approaches. Gaussian filtering and NSAP effectively suppress noise; however, their PSNR and SSIM values remain moderate, suggesting that noise reduction alone is insufficient to enhance subtle pulmonary nodules. CLAHE-based techniques consistently outperform conventional methods due to their ability to adapt contrast locally. Among these, CLAHE optimized using Genetic Algorithm (GA) and Particle Swarm Optimization (PSO) shows noticeable improvement, but their performance is surpassed by CMA-ES optimization. The proposed CMA-ES with a task-specific composite objective function of optimized CLAHE achieves the highest PSNR of 53.41 dB and SSIM of 0.99 while yielding the lowest MSE of 0.31 and NRMSE of 0.01. These results demonstrate that the proposed optimization strategy provides the best trade-off between contrast enhancement and structural preservation. Consequently, CMA-ES with a task-specific composite objective function of optimized CLAHE is selected as the final enhancement technique for all subsequent experiments.

### 4.6. Baseline Classification Results Without Enhancement

This subsection presents the baseline classification performance of the investigated deep learning models when trained on raw CT images, without applying any intensity enhancement or contrast normalization techniques. Establishing this baseline is essential to quantify the inherent limitations of deep learning models operating directly on unprocessed CT data and to provide a clear reference point for evaluating the effectiveness of the proposed enhancement framework.

#### 4.6.1. Model-Wise Baseline Performance

The baseline classification results for ResNet-50, EfficientNet-B0, and InceptionV3 are summarized in Table 8. Among the evaluated architectures, ResNet-50 achieves the highest overall performance, with an accuracy of 95%, a precision of 97%, and an F1-score of 96.5%. This relatively strong baseline performance can be attributed to the residual learning mechanism of ResNet-50, which facilitates stable gradient propagation and enables effective extraction of hierarchical features even in the presence of moderate noise. EfficientNet-B0 achieves a baseline accuracy of 91% with a precision of 89% and a recall of 85%. While its lightweight architecture and compound scaling strategy provide computational efficiency, its reduced depth and parameter count limit its ability to capture subtle textural variations present in low-contrast pulmonary nodules when trained on raw images. InceptionV3 exhibits the weakest baseline performance, with an accuracy of only 79% and balanced precision and recall values of 79%. Despite its multi-scale feature extraction design, the model struggles to learn discriminative representations from raw CT scans due to pronounced intensity variability and insufficient local contrast, which reduce the effectiveness of parallel convolutional branches.

Overall, the baseline results reported in Table 8 indicate that, although modern CNN architectures can achieve reasonable performance on unprocessed CT data, their diagnostic reliability remains limited, particularly in terms of sensitivity to small or low-contrast nodules.

#### 4.6.2. Discussion of Baseline Failure Modes

A closer examination of the baseline results reveals several consistent failure patterns across all evaluated models. The most prominent limitation is the reduction in recall, particularly for EfficientNet-B0 and InceptionV3, indicating that a significant number of malignant nodules are missed during classification. These false negatives predominantly correspond to nodules with small diameters, weak boundaries, or intensity profiles that closely resemble surrounding lung parenchyma, a challenge also reported in prior studies [7]. The primary cause of these failures lies in the inherent intensity inconsistency and noise present in raw CT images. Variations introduced by different scanners, reconstruction kernels, and acquisition protocols lead to non-uniform contrast and fluctuating Hounsfield Unit distributions. As a result, deep learning models tend to learn scanner-specific intensity patterns rather than anatomy-driven features, which negatively impacts generalization [16]. Furthermore, noise and low local contrast obscure critical morphological cues such as nodule margins, internal texture heterogeneity, and shape irregularities. This makes it difficult for convolutional filters to distinguish nodules from visually similar structures such as blood vessels, airway walls, and partial volume artifacts, which has also been highlighted in earlier CT-based studies [60]. Consequently, feature extraction becomes unreliable, particularly in early network layers where contrast-sensitive edge detection is crucial.

These baseline limitations clearly demonstrate that relying solely on raw CT images is insufficient for robust lung nodule classification. The observed degradation in recall and overall accuracy provides strong motivation for incorporating adaptive image enhancement techniques to normalize intensity distributions, improve contrast, and enhance diagnostically relevant structures prior to deep learning-based analysis [17].

### 4.7. Classification Results with Optimized CLAHE

This section presents the classification performance of the proposed deep learning models after applying the Modified CMA-ES-optimized CLAHE enhancement technique. The objective of this evaluation is to quantify the extent to which optimized contrast enhancement improves discriminative learning, reduces misclassification, and enhances robustness compared to baseline performance obtained from raw CT images. The quantitative classification results obtained after applying the optimized CLAHE preprocessing are summarized in Table 9. A clear and consistent improvement is observed across all evaluated deep learning architectures, confirming the positive impact of enhancement on downstream classification performance.

Among the three models, ResNet-50 demonstrates the strongest overall performance, achieving an accuracy of 99.0%, a precision of 98.88%, and an F1-score of 99.0%. These results indicate that the residual learning framework of ResNet-50 effectively leverages the enhanced contrast and preserved structural information to extract highly discriminative features from pulmonary nodule regions. EfficientNet-B0 also exhibits substantial gains following enhancement, particularly in terms of sensitivity. As reported in Table 9, EfficientNet-B0 achieves the highest recall of 99.10%, indicating an excellent ability to detect malignant nodules and minimize false negative predictions. This characteristic is especially desirable in clinical screening scenarios, where missing a malignant case carries significant diagnostic risk. InceptionV3, which showed comparatively weaker baseline performance, benefits markedly from the optimized enhancement. After CLAHE optimization, InceptionV3 reaches an accuracy of 96.96% and an F1-score of 96.9%, reflecting improved multi-scale feature learning once local contrast inconsistencies are mitigated. Overall, the enhanced results presented in Table 9 demonstrate that contrast-optimized input representations enable all three architectures to operate closer to their full representational potential, with ResNet-50 emerging as the most reliable and balanced model.

### 4.8. Inference Time Analysis

The average inference time of the proposed ResNet-50-based framework was evaluated on an NVIDIA RTX 3090 GPU. For a single CT image resized to 224×224, the average test-time inference was approximately 15 ms per image, including preprocessing and classification. The optimized CLAHE parameters are determined offline only once; therefore, the preprocessing stage adds negligible overhead during deployment. These findings suggest that the proposed method is computationally practical for near real-time clinical screening applications.

### 4.9. Performance Gain over Baseline

To assess the effectiveness of the proposed enhancement strategy, the enhanced results are directly compared with baseline performance obtained from raw CT images, as reported in Table 8. The comparison reveals substantial absolute and relative performance gains across all evaluated models. Specifically, ResNet-50 exhibits an accuracy improvement of approximately 4.7%, increasing from 95% at baseline to 99.0% after enhancement. EfficientNet-B0 shows an even larger improvement of approximately 5.9%, while InceptionV3 demonstrates the most pronounced gain, with an accuracy increase of approximately 17%. These improvements are not limited to accuracy alone. Gains in recall and F1-score are particularly noteworthy, indicating that optimized CLAHE primarily strengthens the models’ ability to detect subtle nodules rather than merely improving confidence in already easy-to-classify cases. This behavior confirms that contrast optimization enhances diagnostically relevant structures, allowing networks to better distinguish nodules from surrounding lung tissue and vessel-like patterns. The observed gains are in line with prior reports that improved input normalization can enhance downstream classification performance.

#### Discussion on Statistical Significance

Although statistical hypothesis testing was not performed, consistent improvements were observed across all evaluated models and metrics. The performance gains are substantial compared to baseline results, suggesting that the improvements are meaningful rather than incidental.

Future work will include rigorous statistical validation using multiple runs and hypothesis testing.

### 4.10. Error Analysis After Enhancement

Despite the strong overall performance achieved after enhancement, a small number of misclassifications persist. Detailed inspection of misclassified cases reveals that remaining errors are predominantly associated with very small nodules, nodules exhibiting extremely low contrast, and juxta-vascular nodules that are closely attached to blood vessels. Similar challenging cases have also been discussed in prior studies. False positive errors typically arise from vascular bifurcations or airway cross-sections that retain nodule-like appearances even after enhancement. Conversely, residual false negatives mainly involve nodules near the spatial resolution limit of CT imaging or those affected by motion artifacts and partial volume effects. Importantly, comparison with baseline confusion patterns reveals a substantial reduction in false negative cases across all models, particularly for ResNet-50 and EfficientNet-B0. This reduction is clinically significant, as false negatives correspond to missed cancer cases. The optimized CLAHE preprocessing effectively improves the visibility of weak boundaries and low-contrast nodules, enabling the networks to correctly identify cases that were previously overlooked.

### 4.11. Ablation Study

To better understand the individual contribution of each major component in the proposed framework, a comprehensive ablation study is conducted. This type of component-wise analysis is commonly used to quantify the contributions of different modules in deep learning frameworks [61]. The objective of this analysis is to isolate the impacts of (i) image enhancement, (ii) CutMix data augmentation, and (iii) transfer learning on overall classification performance. By systematically enabling and disabling these components, their influence on learning behavior, robustness, and diagnostic accuracy is quantitatively and qualitatively assessed. All ablation experiments are performed using identical dataset splits, training configurations, and evaluation metrics to ensure a fair and unbiased comparison.

Table 10 presents the quantitative ablation results for ResNet-50 under different component settings.

The ablation results clearly demonstrate the contribution of each component in the proposed framework. Compared with the baseline configuration, optimized CLAHE enhancement improves all evaluation metrics, confirming the importance of contrast normalization for reliable feature extraction. CutMix augmentation also contributes to performance improvement by enhancing generalization and reducing overfitting. The full proposed model, which combines enhancement, CutMix, and transfer learning, achieves the best overall performance with 99.00% accuracy and 99.00% F1-score. In contrast, removing transfer learning causes a clear drop in performance, highlighting the critical role of pretrained feature initialization in the proposed framework.

#### 4.11.1. Effect of Image Enhancement Only

The first ablation experiment evaluates the influence of image enhancement independently, without the application of CutMix augmentation. In this setting, models are trained using CT images enhanced with the optimized CLAHE technique, while no advanced data augmentation is applied beyond basic preprocessing. The results demonstrate that enhanced images consistently outperform raw CT images across all evaluated architectures. Compared with baseline models trained on unprocessed scans, enhanced input models achieve noticeably higher accuracy, precision, and recall. The most pronounced improvement is observed in recall, indicating a reduction in missed malignant nodules. This performance gain can be attributed to improved local contrast and clearer boundary delineation introduced by the optimized CLAHE. Enhanced images exhibit more distinguishable nodule margins and internal texture patterns, which facilitate more effective feature extraction by convolutional layers. Importantly, this improvement is achieved without modifying network architectures or increasing model complexity, confirming that input-level optimization alone has a substantial impact on diagnostic performance. These findings validate that intensity normalization and contrast enhancement are critical prerequisites for reliable lung nodule classification, and that raw CT images are insufficient for optimal deep learning performance due to noise and scanner-induced intensity variability.

#### 4.11.2. Effect of CutMix Data Augmentation

The second ablation study examines the contribution of CutMix augmentation by comparing enhanced models trained with and without CutMix. In this experiment, optimized CLAHE preprocessing is applied in both cases, ensuring that the augmentation strategy is the only varying factor [56]. Models trained with CutMix demonstrate consistently improved generalization behavior. Quantitatively, CutMix leads to higher recall and F1-scores, particularly for EfficientNet-B0 and InceptionV3. Qualitative analysis of training curves reveals smoother convergence and a smaller gap between training and validation performance, indicating reduced overfitting [62]. CutMix encourages the network to learn from partially mixed anatomical structures rather than relying on small, highly discriminative regions. This discourages memorization and promotes contextual reasoning, which is especially important in lung CT imaging where nodules often occupy only a small fraction of the image. As a result, the models become more robust to positional bias, noise, and intensity fluctuations [28]. Overall, this ablation confirms that CutMix acts as an effective regularizer, improving sensitivity to malignant nodules while stabilizing the training process. Its benefits are most evident in recall-oriented metrics, which aligns well with clinical priorities where minimizing false negatives is critical [1].

#### 4.11.3. Effect of Transfer Learning

The final ablation study investigates the importance of transfer learning by comparing models initialized with ImageNet-pretrained weights against models trained from scratch using random initialization. This comparison is conducted using enhanced images and CutMix augmentation to isolate the effect of pretraining [31]. Pretrained models consistently converge faster and achieve higher final accuracy than their randomly initialized counterparts. Models trained from scratch exhibit slower convergence, higher initial loss values, and increased susceptibility to overfitting, particularly for deeper architectures such as ResNet-50 and InceptionV3 [63]. The advantage of transfer learning arises from the reuse of generic low- and mid-level visual features learned from large-scale datasets. These features, including edge detectors, texture filters, and shape primitives, transfer effectively to medical imaging tasks despite differences between natural and medical image domains. Fine-tuning allows higher layers to adapt these representations to lung-specific anatomical patterns and pathological structures [27].

This ablation study demonstrates that transfer learning is not merely a convenience but a critical component for achieving strong performance on limited medical datasets. Without pretraining, models struggle to learn robust representations, leading to inferior accuracy and reduced generalization capability [61].

### 4.12. Confusion Matrix Analysis

To further investigate the classification behavior of the proposed framework beyond aggregate performance metrics, confusion matrix analysis is performed for all three deep learning architectures trained using the Modified CMA-ES-optimized CLAHE preprocessing. Confusion matrices provide a detailed breakdown of true positives (TP), true negatives (TN), false positives (FP), and false negatives (FN), thereby offering valuable insight into class-wise prediction reliability and potential diagnostic risks.

#### 4.12.1. ResNet-50 Confusion Matrix

**Figure 8:**
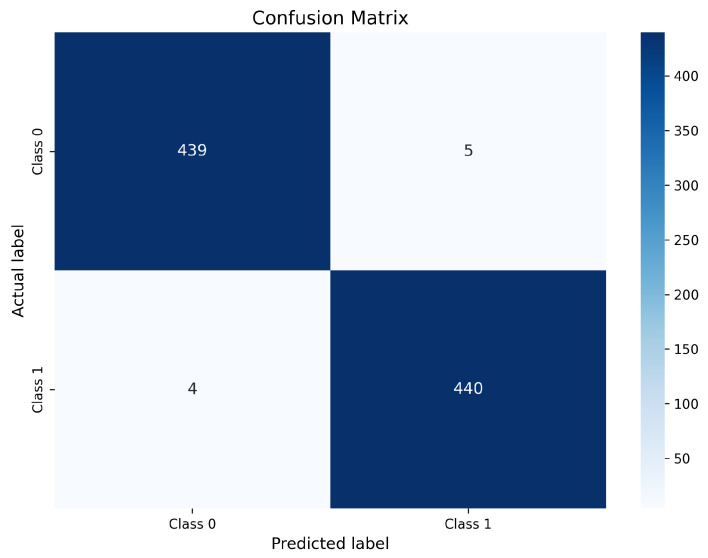
Confusion matrix of ResNet-50 trained with optimized CLAHE.

The confusion matrix obtained for the ResNet-50 model is illustrated in Figure 8.

As shown in Figure 8, the ResNet-50 model correctly classifies 439 non-nodule samples as negative (TN) and 440 nodule samples as positive (TP). Only 5 non-nodule cases are incorrectly predicted as nodules (FP), while merely 4 nodule cases are misclassified as non-nodules (FN). This distribution demonstrates an excellent balance between sensitivity and specificity. The extremely low false negative count is particularly important in lung cancer screening, since false negatives correspond to missed malignant nodules that may delay diagnosis and treatment. The small number of FP further indicates strong specificity, reducing unnecessary follow-up examinations and radiologist workload. These results confirm that ResNet-50 effectively exploits the enhanced contrast and preserved structural details produced by optimized CLAHE, enabling highly reliable discrimination between nodules and non-nodules.

#### 4.12.2. EfficientNet-B0 Confusion Matrix

**Figure 9:**
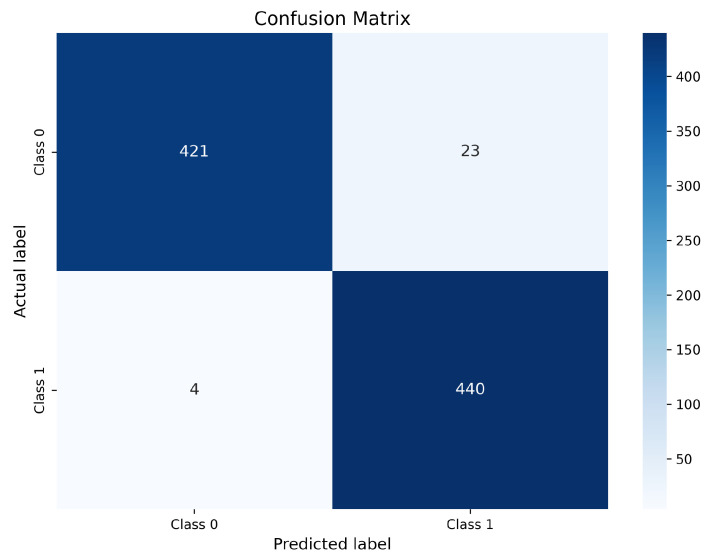
Confusion matrix of EfficientNet-B0 trained with optimized CLAHE.

The confusion matrix corresponding to EfficientNet-B0 is presented in Figure 9.

Figure 9 shows that EfficientNet-B0 correctly identifies 421 non-nodule samples (TN) and 440 nodule samples (TP). The model produces 23 false positive predictions and only 4 false negative predictions. The very small FN count indicates excellent sensitivity and confirms EfficientNet-B0’s strong ability to detect malignant nodules. However, the higher FP count compared with ResNet-50 suggests a slight reduction in specificity. This behavior reflects a conservative detection tendency, where the model prefers to flag ambiguous regions as suspicious rather than risk missing malignant cases. From a clinical perspective, such behavior may lead to additional benign alerts, but it is generally acceptable in screening settings where minimizing missed cancers is prioritized.

#### 4.12.3. InceptionV3 Confusion Matrix

**Figure 10:**
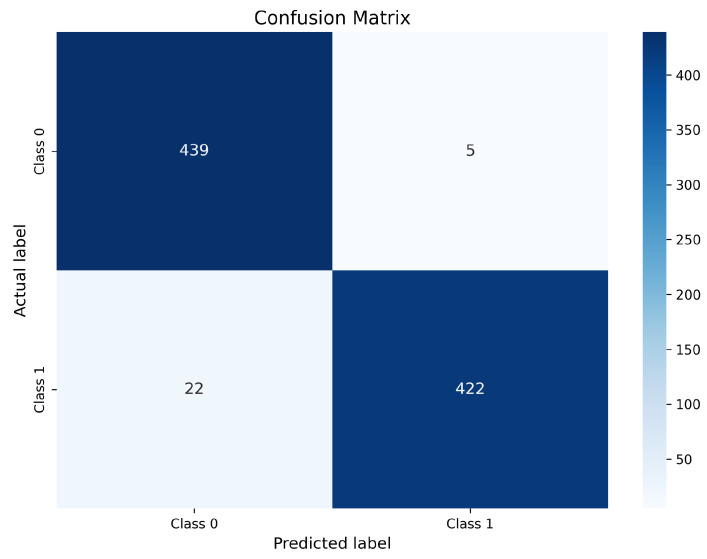
Confusion matrix of InceptionV3 trained with optimized CLAHE.

The confusion matrix for the InceptionV3 model is shown in Figure 10.

InceptionV3 correctly classifies 439 non-nodule samples (TN) and 422 nodule samples (TP). However, it produces 5 false positive predictions and a comparatively higher number of false negatives (22 cases). The increased FN count indicates that InceptionV3 remains less sensitive to subtle or low-contrast nodules, even after enhancement. Although its multi-scale feature extraction capability is beneficial, it appears less effective than residual and compound-scaled architectures in leveraging contrast-optimized inputs for fine-grained nodule discrimination. Overall, the confusion matrix analysis confirms that optimized CLAHE preprocessing substantially improves class-wise prediction reliability across all evaluated models. Among the three architectures, ResNet-50 exhibits the most balanced performance with the lowest combined FP and FN counts, making it the most clinically robust model. EfficientNet-B0 achieves comparable sensitivity with slightly higher FP, while InceptionV3 demonstrates comparatively weaker consistency due to its higher false negative rate. Most remaining misclassifications are associated with very small nodules, low-contrast lesions, and juxta-vascular nodules, which remain challenging even after enhancement; similar difficult cases are also discussed in prior studies.

### 4.13. Training Dynamics and Convergence

**Figure 11:**
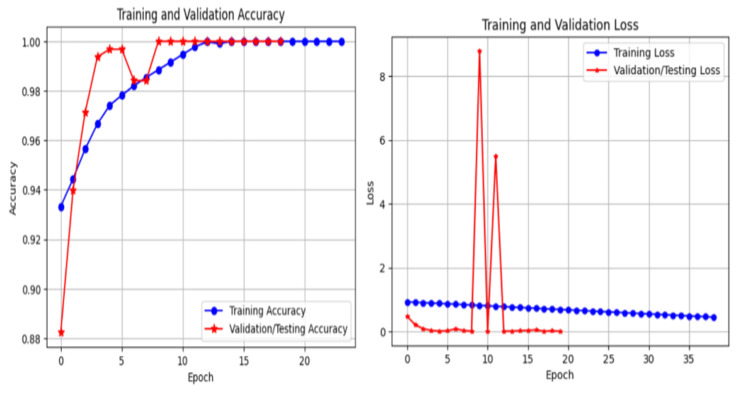
Training and validation accuracy and loss curves for ResNet-50 using optimized CLAHE and CutMix.

**Figure 12:**
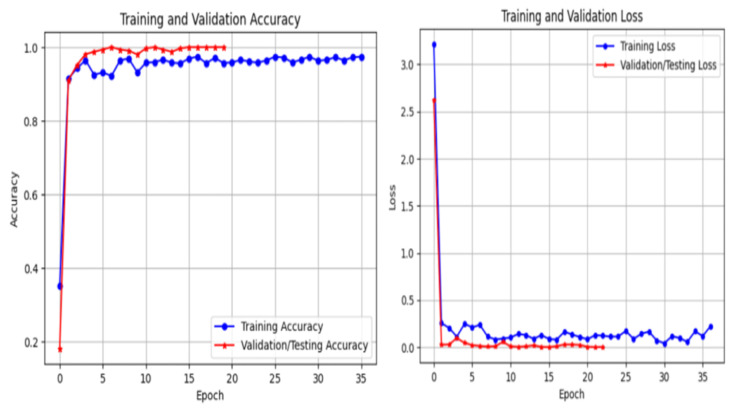
Training and validation accuracy and loss curves for EfficientNet-B0 using optimized CLAHE and CutMix.

**Figure 13:**
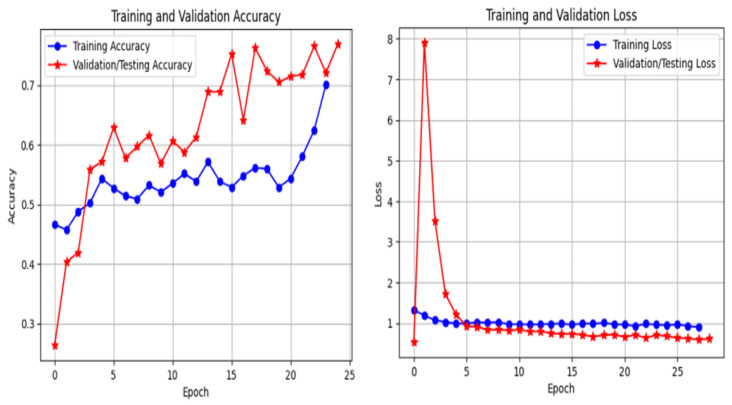
Training and validation accuracy and loss curves for InceptionV3 using optimized CLAHE and CutMix.

The training behavior of the proposed framework is analyzed through training and validation accuracy and loss curves for ResNet-50, EfficientNet-B0, and InceptionV3, as illustrated in Figure 11, Figure 12 and Figure 13. These curves provide insight into convergence speed, optimization stability, and potential overfitting.

As shown in Figure 11, ResNet-50 demonstrates smooth and stable convergence. The training and validation accuracy curves increase rapidly during the early epochs and remain closely aligned throughout training, indicating strong generalization and minimal overfitting. Similarly, the loss curves decrease steadily and converge to low values, reflecting stable optimization. EfficientNet-B0 (Figure 12) exhibits rapid convergence, achieving high validation accuracy within a small number of epochs. Although minor fluctuations are observed, the gap between training and validation curves remains limited, suggesting effective regularization and good generalization. In contrast, InceptionV3 (Figure 13) shows slower convergence and higher variability, particularly in the early training phase. The loss curve exhibits larger oscillations, indicating greater sensitivity to optimization and data variability. Nevertheless, convergence is eventually achieved. Overall, these results confirm that optimized CLAHE preprocessing combined with CutMix augmentation leads to stable and efficient training, with ResNet-50 providing the most consistent learning behavior.

### 4.14. Model Complexity and Computational Efficiency

Model complexity and computational efficiency are important considerations for practical deployment in clinical environments. EfficientNet-B0 contains significantly fewer parameters than ResNet-50 and InceptionV3, making it the most lightweight model in this study. Despite having a higher parameter count, ResNet-50 achieves the best accuracy–complexity trade-off by delivering superior diagnostic performance with moderate computational cost. InceptionV3 has higher architectural complexity but does not provide corresponding performance gains. These observations indicate that EfficientNet-B0 is suitable for resource-constrained systems, whereas ResNet-50 is preferable when higher diagnostic accuracy is prioritized.

### 4.15. Comparison with Existing Methods

The proposed framework is compared with recent LUNA16-based studies reported in the literature. Most existing works focus on lung nodule detection and report performance using sensitivity, Free-Response Receiver Operating Characteristic (FROC), and Competition Performance Metric (CPM). In contrast, the present study addresses a classification task using candidate-level patches rather than full detection pipelines.

Although direct comparison with detection-based methods is not strictly equivalent, recall (sensitivity) is reported as a proxy for detection performance. The proposed method achieves a recall of 99.1%, making it competitive with respect to recent studies reporting sensitivity values above 95%.

It is important to note that detection frameworks typically incorporate candidate generation and false positive reduction stages, enabling evaluation using FROC and CPM. Since the current work focuses on classification after candidate extraction, these metrics are not directly applicable.

Future work will extend the proposed framework to full detection pipelines and include standardized evaluation using FROC and CPM for direct comparison with state-of-the-art methods.

### 4.16. Robustness Across Intensity Groups

To evaluate robustness against scanner-induced intensity variability, performance is analyzed across low-, medium-, and high-intensity CT scan groups. Without enhancement, model performance varies considerably across these groups. After applying optimized CLAHE, classification accuracy becomes more consistent across all intensity levels, indicating that the proposed enhancement successfully normalizes contrast differences. The experimental results also show improved image quality, better feature visibility, and more reliable classification. The integration of CutMix augmentation further enhances generalization and stabilizes training. ResNet-50 consistently achieves the best overall performance, followed by EfficientNet-B0, while InceptionV3 shows comparatively weaker stability. Most importantly, the framework substantially reduces false negative detections. Similar robustness challenges and the need for intensity-consistent preprocessing have been reported in prior multi-scanner CT studies. From a clinical perspective, reducing false negatives is particularly important in lung cancer screening.

## 5. Conclusions

This study introduced a robust and clinically motivated framework for pulmonary nodule classification in chest CT images, addressing a key challenge in computer-aided diagnosis: scanner- and protocol-induced intensity variability. Rather than increasing architectural complexity, the proposed approach emphasizes adaptive, optimization-driven preprocessing at the input level to improve feature consistency and learning stability.

A key contribution of this work is the formulation of CLAHE parameter selection as an optimization problem using CMA-ES with a task-specific composite objective function. By jointly maximizing PSNR and SSIM while minimizing NRMSE, the method effectively balances contrast enhancement with structural preservation. The quantitative results demonstrate that the optimized CLAHE configuration outperforms conventional enhancement techniques, achieving PSNR values of approximately 53.41dB and SSIM values close to 0.99.

The effectiveness of the proposed preprocessing was validated using three deep learning architectures (ResNet-50, EfficientNet-B0, and InceptionV3) on the LUNA16 dataset. Significant improvements were observed across all models, particularly in sensitivity to small and low-contrast nodules. ResNet-50 achieved the best overall performance with an accuracy of 99.0%, while EfficientNet-B0 achieved the highest recall of 99.10%.

These results demonstrate that improved input standardization enables deep learning models to operate closer to their full representational capacity. The proposed framework shows potential for future clinical applicability by improving robustness and reducing false negative detections, although further validation is required.

A limitation of this study is that all experiments were conducted on the LUNA16 dataset, which represents a relatively homogeneous data source. External validation on independent multi-center datasets was not performed, limiting the strength of generalizability claims. In addition, no clinical validation or reader studies involving radiologists were conducted. Therefore, the practical utility of the proposed framework in real-world clinical workflows remains to be established.

A further limitation is that the proposed framework is evaluated at the classification stage and does not include a full detection pipeline. Therefore, standard detection metrics such as FROC and CPM are not reported.

Future work will focus on validating the framework on multi-center datasets with diverse scanners and acquisition protocols, extending the approach to 3D volumetric analysis, and integrating explainability mechanisms and domain-adaptive learning strategies to further enhance robustness and deployment potential.

## Figures and Tables

**Table 1:** Comparison of intensity correction techniques in lung CT imaging.

Paper ID	Technique	Strength	Limitations
[33]	Histogram Equalization	Improves global contrast and dynamic range.	May amplify noise and non-nodular regions.
[34]	CLAHE	Enhances local contrast and improves feature visibility.	Sensitive to clip limit and tile-size selection.
[35]	Adaptive Median Filtering + CLAHE	Combines denoising and local contrast enhancement.	Multi-stage tuning may blur subtle structures.
[29]	Mean-Variance Normalization	Standardizes feature scales across scans.	Does not correct inter-scan intensity bias.
[36]	Bias Field Correction	Improves harmonization across scanners.	May introduce distortion if over-corrected.
[37]	GAN-Based Intensity Adjustment	Learns scanner harmonization in a data-driven manner.	Risk of hallucinated features and reduced reliability.

**Table 2:** Comparison of lung nodule detection models.

Paper ID	Model Type	Accuracy	Limitations
[41]	2D CNN	∼96%	Limited spatial context and weaker robustness to low contrast.
[29]	Radiomics + SVM	84.6%	Manual feature engineering and limited generalization.
[7]	3D CNN	96%	High computational cost and risk of overfitting.
[33]	ResNet-50	97.9%	Sensitive to dataset-specific intensity distribution.
[42]	VGG-16 + Capsule Network	99.2%	Increased architectural complexity and limited external validation.
[32]	Attention-augmented CenterNet	98%	Sensitive to severe intensity variation and higher inference cost.
[28]	CNN Ensemble	93.6%	Ensemble complexity and limited robustness on small datasets.
[43]	Fuzzy Segmentation + Bi-LSTM	97.7%	Multi-stage pipeline and higher runtime complexity.
[44]	Transformer-Based Model	>95%	Large data requirements and reduced interpretability.

**Table 3:** FP rates reported in lung CT studies.

Paper ID	Method	FPR	Remarks
[6]	Multi-view CNN with candidate merging	∼4 FP/scan @ ∼85% sensitivity	Early deep learning method with high FP burden.
[10]	Two-stage 2D Faster R-CNN with 3-slice ensemble	0.39 FP/scan @ 83.3% sensitivity	Improved refinement through staged detection.
[45]	3D FCN + 3D ResNet FP reducer	1 FP/scan @ 90.5% sensitivity	Volumetric context improves specificity.
[46]	TSND (MSFD-Net + CS-Net)	0.125–8 FP/scan @ 90.59% sensitivity	Robust performance across FP operating points.
[47]	Self-supervised 3D FPN with HS-Specificity		
Net	0.125 FP/scan @ 90.6% sensitivity	Very low FPR using volumetric consistency.	
[32]	Attention-augmented CenterNet	∼0 FP/scan	Excellent benchmark precision, but still intensity-sensitive.

**Table 4:** Dataset splitting percentages and number of CT scans.

Dataset	Splitting Percentage	Number of CT Scans
Training set	70%	622
Testing set	20%	178
Validation set	10%	88
Total	100%	888

**Table 5:** Hardware and software environment used for experiments.

Component	Specification
CPU	Intel Core i9
GPU	NVIDIA RTX 3090 (24 GB VRAM)
RAM	64 GB
Operating System	Ubuntu 20.04
Programming Language	Python 3.8
Deep Learning Framework	TensorFlow 2.x (Keras API)
CUDA/cuDNN	CUDA 11.x/cuDNN 8.x

**Table 6:** Training hyperparameters used in all experiments.

Parameter	Value
Input size	224×224
Batch size	16
Optimizer	Adam
Learning rate	$1\times 10^{-4}$
Epochs	150
Loss function	Binary Cross-Entropy
Dropout rate	0.5
Transfer learning	ImageNet pretrained

**Table 7:** Quantitative Comparison of Image Enhancement Techniques.

Technique	PSNR	SSIM	MSE	NRMSE
Wavelet	44.44	0.98	2.34	0.01
Retinex	10.71	0.80	5527.34	0.56
CEPIN	17.36	0.92	1194.21	0.26
Gaussian	24.94	0.74	208.72	0.11
NSAP	24.35	0.74	239.01	0.12
Histogram Equalization	15.34	0.52	1900.95	0.33
CLAHE + GA	24.35	0.74	239.01	0.12
CLAHE + PSO	26.09	0.86	2.10	0.03
CLAHE + CMA-ES	36.67	0.96	14.10	0.01
**CMA-ES with a task-specific composite objective function (Proposed)**	**53.41**	**0.99**	**0.31**	**0.01**

**Table 8:** Baseline Model Performance Without Image Enhancement.

Model	Precision (%)	Recall (%)	F1-Score (%)	Accuracy (%)
ResNet-50	97	96	96.5	95
EfficientNet-B0	89	85	87	91
InceptionV3	79	79	79	79

**Table 9:** Comparative Diagnostic Performance with Optimized CLAHE.

Model	Precision (%)	Recall (%)	F1-Score (%)	Accuracy (%)
ResNet-50	**98.88**	**99.1**	**99.0**	**99.0**
EfficientNet-B0	95.03	**99.10**	97.03	96.96
InceptionV3	98.83	95.05	96.9	96.96

**Table 10:** Ablation Study Showing Contribution of Each Component (ResNet-50).

Configuration	Enh.	CutMix	TL	Accuracy (%)	Precision (%)	Recall (%)	F1-Score (%)
Baseline (Raw CT)	No	No	Yes	95.00	97.00	96.00	96.50
Enhancement only	Yes	No	Yes	97.50	97.80	97.20	97.50
CutMix only	No	Yes	Yes	96.50	97.10	96.00	96.55
Full Model (Proposed)	Yes	Yes	Yes	99.00	98.88	99.10	99.00
No Transfer Learning	Yes	Yes	No	94.00	93.50	93.80	93.65

## Data Availability

The original contributions presented in the study are included in the article; further inquiries can be directed to the corresponding author.
